# Sports and Exercise by Gender and Odds Ratios of Obesity in Children

**DOI:** 10.3390/sports14040159

**Published:** 2026-04-17

**Authors:** Bernadett Wágner, Petra Halmi, Martina Uvacsek

**Affiliations:** 1 School of Doctoral Studies, Hungarian University of Sports Science, Alkotás u. 42-48, 1123 Budapest, Hungary; wagner.bernadett@tf.hu (B.W.); halmi.petra23@gmail.com (P.H.); 2 Department of Health Sciences and Sport Medicine, Hungarian University of Sports Science, Alkotás u. 42-48, 1123 Budapest, Hungary

**Keywords:** physical activity, obesity prevention, sport participation, health promotion

## Abstract

This study aimed to collect survey data on physical activity and organized sport participation among children across different weight categories. Using online data collection, 906 parents provided information about 1002 children (age = 10.9 ± 2.5 years, body height = 150.2 ± 16.4 cm, and body mass = 42.4 ± 15.8 kg). Most children (79%) achieved the recommended 60 min. of moderate-to-vigorous physical activity (MVPA) per day; 50% participated in club sports; and 41% took part in organized sports. Most of them (69%) were in the healthy weight category, 7% were underweight, and 22% of the children were overweight or obese. Participation in sports activities among overweight and obese children was nearly as high as among their peers. Achieving 60 min. of MVPA/day was associated with significantly lower odds (OR = 0.51 CI: 0.30–0.85; *p* < 0.01) of childhood obesity in the total sample. Football was the most popular club and organized sport among boys, while dancing was most preferred among girls. The preferred sports were generally well-suited to the respective weight categories. The relatively high levels of physical activity observed may be explained by daily physical education (PE) classes in schools and governmental support for sports. Our findings suggest that further research is needed to support effective obesity prevention, particularly those examining dietary habits and other lifestyle factors.

## 1. Introduction

Childhood and adolescent overweight and obesity are major global public health concerns, affecting both developed and developing countries worldwide [1,2]. This trend is alarming, given the strong tracking of obesity from childhood into adulthood and its well-documented associations with impaired physical fitness, adverse cardiometabolic outcomes, and reduced quality of life [3].

In Hungary, data from international monitoring systems, such as the Health Behavior in School-aged Children (HBSC) study, indicate that the rate of childhood overweight exceeds the international average, underscoring the urgent need for effective, evidence-based prevention strategies [4]. The development of childhood obesity is multifactorial, resulting from complex interactions between genetic susceptibility, environmental influences, behavioral factors, and socioeconomic conditions [5,6]. According to the latest HBSC study, decreases in regular breakfast consumption and, more generally, in sleep duration hours, along with increases in electronic media use, might be obesogenic factors [4]. Regular physical activity is a cornerstone of obesity prevention, supporting healthy growth and development [7]. Higher levels of moderate-to-vigorous physical activity are associated with favorable body composition, improved cardiovascular and musculoskeletal fitness, enhanced metabolic health, and better psychological well-being in youth. Accordingly, international guidelines emphasize the importance of promoting physical activity while limiting sedentary time [8]. However, despite these recommendations and the implementation of school-based initiatives such as daily physical education, overall physical activity levels among children and adolescents remain below recommended levels in many countries, including Hungary [4]. Current prevention strategies have not led to overall lifestyle changes; the introduction of daily PE cannot compensate for increased sedentary time, reduced sleep duration, or inappropriate eating habits.

Participation in organized sports is a widely adopted and effective strategy for promoting physical activity in young people. Sports club membership is consistently associated with higher levels of exercise and activity, lower sedentary time, and improved fitness [9,10]. Beyond overall activity volume, the type and structure of physical activity appear to be critical determinants of whether children and adolescents meet physical activity recommendations [8]. Evidence from a large international study indicates that participation in organized and more vigorous activity types, particularly team sports, contributes substantially to higher overall physical activity levels among youth compared with unstructured or low-intensity activities [11]. Similarly, a study of 10-year-old children in Norway identified specific physical activities associated with higher cardiorespiratory fitness, with team sports such as handball, volleyball, and basketball showing positive associations, whereas activities like frisbee and dodgeball were less beneficial [12]. These results underline the importance of considering activity type and preference when designing interventions to promote health-enhancing physical activity in children.

In addition to individual and behavioral factors, the role of environmental characteristics in shaping children’s physical activity patterns is crucial. The type of built environment influences overall physical activity, with suburban children generally exhibiting the highest activity levels, followed by rural and urban children [13,14]. Rural children, however, tend to spend more time outdoors in unstructured play compared with their urban peers, particularly under the age of 13 years [11]. Furthermore, parental perceptions of neighborhood characteristics—including safety, street connectivity, and access to recreational spaces—have been shown to influence children’s location-specific physical activity, highlighting the importance of both objective and subjective environmental factors [15].

Parental education, parental weight status, and support for physical activity have all been linked to childhood overweight and obesity, although the strength and direction of these associations vary across countries and socioeconomic contexts [16,17]. In high-income countries, higher parental education is generally associated with a lower prevalence of childhood overweight, while parental encouragement and involvement in sport are key facilitators of children’s sustained participation in physical activity [17,18].

Despite extensive research on childhood obesity and physical activity, limited information is available on how overall physical activity, club sport, organized sport participation and activity with parents differ by gender. The focus of this study was to assess overall physical activity, participation in club and organized sports, and activity with parents, among school-aged children and to compare these behaviors across different weight status categories. A secondary aim was to assess the odds associated with different factors affecting physical activity and the development of obesity in children. Based on previous HBSC studies conducted in Hungary, we hypothesized that the main cause of obesity is the lack of physical activity.

## 2. Materials and Methods

### 2.1. Participants

The study was conducted in accordance with the declaration of Helsinki; prior to any data collection, ethical approval was received from the Hungarian University of Sports Science (MTSE-OKE KEB/08/2023). This study was a cross-sectional survey study and was conducted between 2023 spring and 2025 spring using social media platforms and internet correspondence. The research project was sent to 1247 principals of primary schools in Budapest and in Pest County, who were asked to share it with parental groups. The respondent parents were mostly mothers (85%), 12% fathers, and 3% grandparents or relatives. The first page contained the description and the aim of the research data collection and the inclusion criteria. All the parents who had a primary school aged (6–15 years old) child were able to fill in the form voluntarily. Bias was avoided by the targeted increased sample size and the IP address check and by diversity of residencies. After cleaning the data from the online collection, the final sample provided information about 1002 children (10.9 ± 2.5 years old). The 1002 responses (514 boys and 488 girls) were collected from 906 different IP addresses. Informed consent was the first part of the questionnaire that was obtained from all participants prior to answering any questions. Altogether, only 5 parental answers were excluded due to missing information or errors.

### 2.2. Questionnaire

The questionnaire was completed with the Qualtrics program and contained 35 questions. Altogether 13 questions were made for the respondent guardians, and 22 questions were about child’s behavior or physical variables. Parents filled in this self-report questionnaire that captured parents’ demographic and their child’s data. In this report, we analyze the demographic variables and eight questions connected to physical activity. The questionnaire was compiled using the global matrix (GM) 4.0 indicators and definitions. The GM methods and physical activity indicators benchmarks for children and adolescents were published previously [19]. As an overall physical activity indicator, we wanted to know what percentage of children meet the global health-related physical activity recommendation. The question was “Do you think your child accumulates at least 60 min. of moderate-to-vigorous physical activity per day on average?”—“Yes/No”. We used recommended questions for the organized sport and physical activity, and for family indicators. The question for club sport was “Does your child participate in club sports?”—“Yes/No”—“If yes, what kind of sport?”, “Does your child participate in organized sport? (in school or afterschool)”—“Yes/No”—“If yes, what kind of organized sport?”, “Do you do physical activity together with your child?”—“Yes/No”—“If yes, what kind of activity?”, and “Do you achieve the recommended 150 min/week of moderate-to-vigorous physical activity for adults?”—“Yes/No”.

### 2.3. Statistical Analysis

After calculating the body mass index, the weight status categories (underweight, healthy, overweight, and obese) were made according to the World Health Organization (WHO) age- and gender-specific reference [3].

The relationships between being obese and moderate-to-vigorous physical activity (MVPA) and sport participation and parental physical activity behavior and residency were assessed using regression analysis. Majority of the investigated variables were binary except the residency. We created from all residency locations based on two type categories. The Capital of Hungary, county seats, and towns comprised first group, and municipality, village, and farm comprised the second group. Data was analyzed using TIBCO 14.00.15. Statistics and IBM SPSS Statistics 29.0. Descriptive statistics and frequency tables were used to describe demographic characteristics of the children and parents. Chi-square tests were used to compare different participation rates of groups. Binary logistic regression analysis was used to analyze the associations between obesity and different factors affecting physical activity and for the odds ratios (OR) and 95% confidence intervals (CI).

## 3. Results

The characteristics of the children who participated in the study are presented in Table 1. Most of the parent-respondents were mothers (85%), and the majority (73%) of the families lived in towns, county seats, or in the capital of Hungary. Half of the children were male (51%) with a mean age of 10.7 ± 2.5 years. There were no significant differences in age or in body height, body mass, or body mass index (BMI) between genders. According to BMI percentile cut-offs published by the WHO, most children (69%) were in the healthy weight category, 7% were underweight, and 22% were overweight or obese. There were no significant differences in weight category rates by genders.

The participation rates of children in physical activity categorized by gender and weight classifications are presented in Table 2. On average, more than two-thirds of all the children achieved the recommended 60 min. MVPA/day. There were no significant differences by gender or weight classification; however, the highest rate was observed among children with a healthy weight, while the lowest rate was found among obese children. Obese children did not meet the recommendation in 29% of cases, which is significantly higher than in underweight (*p* < 0.04) or healthy (*p* < 0.02) groups. Across all sports and exercise activities, participation rates were slightly lower among females; however these differences were not statistically significant. When comparing different types of sports and exercise the highest participation rates were observed for activities with parents followed by club sport participation. Participation in club sports and engaging in activities with parents were at similar levels across weight categories. Obese children participated significantly less (*p* < 0.05) in organized sports compared to underweight children.

In Table 3, we present the odds ratios of obesity for various factors that may influence the physical activity of children. Achieving a minimum of 60 min. of moderate-to-vigorous physical activity per day and participation in club sports or other organized sports are frequently investigated factors. Parental physical activity, or physical activity performed together with parents, is less frequently studied but remains an important factor, while family residency is rarely investigated. For the total sample presented in Table 3, all investigated factors may reduce the possibility of obesity, as all odds ratios in the first column are below 1 (OR < 1) across all categories; however, only one association reached statistical significance. For most of the investigated factors, the confidence intervals were wide, indicating that the associations were unclear and that additional influencing factors may exist. Only achieving 60 min. of MVPA per day was significant (*p* < 0.01) in the total sample and among males. However, this association could not be confirmed in females when analyzed separately. Another significant factor (*p* < 0.01) that might reduce the odds of obesity was living in a highly populated residential area, but this effect was observed only in males. Participation in club sports and organized sports was not statistically significant; nevertheless, the frequency of these activities was 3.0 ± 1.2 or 2.4 ± 1.5 times per week respectively, in the overall sample.

The preferred sports, exercises, and physical activities by gender and weight status are presented in Table 4. The table includes only those sports and activities that reached a minimum prevalence of 10% in the different subgroups. In both club and organized sports, football was the most preferred activity among boys regardless of weight status. Martial arts are also among the most frequently reported sports for boys across all BMI categories. Handball was particularly preferred by overweight boys, whereas kayaking and canoeing were more frequent among underweight or healthy boys. Organized sports activities showed greater variety. Among obese boys, after football, working out in gym and table tennis were the most frequently chosen activities, while overweight boys also reported athletics as a preferred option. According to girls’ responses, the most popular sport and exercise activity today is dancing, both as an organized sport and as a club sport. Overweight and obese girls preferred handball; however, no single organized sport activity reached a prevalence of 10% among obese girls, as they reported a wide range of different activities. Following handball, basketball also appeared as a frequently reported activity, whereas gymnastics was preferred exclusively by underweight girls.

## 4. Discussion

The primary focus of this study was to assess overall physical activity, participation in club and organized sports, and activity with parents among school-aged children and to compare these behaviors across different weight status categories. A further aim was to examine the odds associated with various factors influencing physical activity and the development of obesity in children. To our knowledge, children’s physical activity participation rates stratified by weight status have rarely been investigated.

According to a systematic review, Hungary has a high prevalence of obesity among children and adolescents in Eastern Europe, with a rate of 6.3% was almost identical to that reported in Bulgaria (6.29%) and Romania (6.46%). Only Southern Europe showed a higher prevalence compared to Eastern Europe [2]. Based on our results, the obesity rate has increased to 8%, particularly among males. However, the methodology of these commonly used questionnaires may have limitations, as overweight and obesity are assessed using BMI alone. The combined prevalence of overweight and obesity in our study was 22%, which is consistent with the findings of the HBSC study and is close to another Hungarian data collection reporting a prevalence of 23% [4,20].

In our study, 79% of children and adolescents achieved the recommended minimum of 60 min. of daily moderate-to-vigorous physical activity, which is higher than the rates previously published by Uvacsek et al. (2024) [16] and in the HBSC global report [4]. In the Global Matrix 4.0 report, the average proportion across participating countries ranged from 27–33% [21]. Our findings are also consistent with results from an HBSC study conducted in Slovakia, where 66% of adolescents met the PA recommendation [22]. Boys typically achieve the recommended level of physical activity at a higher prevalence than girls [4,22,23]; however, in our study, the gender difference was not significant. One possible explanation is that, in Hungary, five PE sessions per week, each lasting 45 min. (one per weekday) were gradually introduced in all schools starting in 2012. Another explanation may relate to methodological differences: in HBSC data collection; children complete questionnaires independently in an online format and may often underestimate their own physical activity. In contrast, the higher prevalence observed in our study may be overestimated due to parental social desirability bias.

We found that 50% of children participated in club sports and 41% participated in organized sports, with no statistically significant gender difference. Participation in organized sports was slightly higher than that reported in a previous Hungarian data collection (40%) [24]. Similar results were found in Slovakia, with 41% overall participation, and higher rates were reported in Poland, with 54% overall participation based on self-reported HBSC data. In Australia, 46% participation in both club and school sports was reported based on a children’s lifestyle survey [22,25,26].

In our study, parent–child joint physical activity was reported by 71% of participants, with no significant difference between genders. This proportion is higher than that reported in Slovakia (46%) and in Poland, where the average was 40%. Parent–child joint physical activity is rarely investigated in the literature; instead, most studies focus on parental support and the influence of parental behavior on children’s physical activity. The specific types of parent–child joint activities were described in a previously published study, in which cycling, walking, and playing football were identified as the most common activities [16].

Our findings indicate that achieving MVPA > 60 min/day is associated with a reduced likelihood of obesity among males. Participation in organized sports has been shown to increase the likelihood of achieving the recommended levels of physical activity [27]. In our study, a high proportion of both genders met the recommended level of physical activity (81% vs. 77%; however, the odds ratio was significant only for boys. The lack of significant and clear associations may be explained by unexamined variables, such as the time and duration of participation in sports or organized sports activities, which may act as important cofounding factors.

Numerous studies have investigated the role of urban lifestyle, the urban environment, and the surroundings of primary schools in relation to childhood obesity [14]. Our finding that an urban environment may be supportive for males is consistent with a longitudinal study by Wolch et al. (2010) [28]. That study reported that greater park availability within 500 m radius of the home was significantly associated with a lower attained BMI in late adolescence, and access to recreational programs within the 10 km buffer zone was inversely related to BMI outcomes. Urban environments may therefore function as protective factors against childhood overweight when they provide structured and accessible opportunities for physical activity [29].

Gender differences in physical activity levels and preferred sport activities are frequently reported in the literature. Cross-sectional studies conducted in Serbia, Norway, and Spain have shown that sport choice is strongly influenced by gender, with boys tending to prefer team sports, while girls are more oriented toward individual sports [12,30]. These studies also identified football as the most popular sport among boys, which is consistent with our findings [31]. Frömel et al. [32] reported similar findings among Czech and Polish boys, indicating that football is the most preferred sport. Martial arts have rarely been mentioned as popular activities in previous studies, except for a Spanish study in which wrestling was mentioned as a preferred activity among boys [30]. In Hungary, martial arts such as judo, wrestling, and boxing are particularly popular, due to the country’s successful participation in the Olympic Games and because martial arts are included in the Hungarian PE curriculum.

Girls preferred activities such as dancing, rhythmic gymnastics, skating, and water sports in a Spanish data collection [30]. These activities are predominantly individual and non-contact sports, which is consistent with our findings showing that dance-related activities are the most preferred forms of physical activity among girls [33]. The lack of relevant organized sport participation among obese girls may be explained by the limited availability of inclusive sport programs, and this issue needs to be investigated in future.

In our opinion, the preferred sports were generally appropriate for the different weight categories. The club and organized sports mentioned above may play an important role in obesity prevention. Activities such as dancing and martial arts involve short bouts of high intensity movement, and this kind of physical activity may contribute to a reduction in adipose tissue. The diversity of these movements may also contribute to sustained participation in sports. According to recent research, integrative neuromuscular training and mind–body fitness might be useful in obesity prevention by offering alternative forms of exercise that provide a feasible and safe exercise experience [34,35].

The present study has several limitations. First, the questionnaire used was not a validated instrument which might limit our results and conclusions, and it could also reduce the replicability of methods and promote bias. Furthermore, the data may reflect only a short period of physical activity behavior in school children. Second, data collection was conducted online which may have further affected data accuracy. In addition, several factors that could influence children’s participation in physical activity and sport were not assessed. Specifically, the exact time spent on physical activity or the duration of participation in sports or organized physical activities was not analyzed. Environmental obesogenic factors, and parental overestimation due to social desirability bias, as well as dietary habits, sedentary behavior, and sleep patterns were also not investigated in the present study.

This study has some strength—the large sample size, and the diversity of data by locations and parental education level—and these might increase our knowledge about attitude of adults who rising children in Hungary. Future research in this field should be multidisciplinary, including the psychological, nutritional, and socio-economic background, to understand the reasons.

## 5. Conclusions

The overall physical activity, and the club and organized sport participation among children increased in this sample in both genders. The overweight and obese children had almost the same participation rates in sport and physical activities as children in healthy weight group. Achieving 60 min. MVPA/day reduced the possibility of childhood obesity in the total sample. Gender differences in preferred sport activities were reported: football was the most popular club and organized sport in boys and dancing was the most preferred in girls. The preferred sports were mostly suitable for the weight categories. The increased physical activity might be explained by the introduction of daily PE in schools and the governmental support of sport. Our results suggest that increased physical activity alone is not enough effective; other lifestyle intervention studies are needed which investigate the obesogenic environment and behavior for successful obesity prevention.

## Figures and Tables

**Table 1:** Characteristics of children.

Children	Total Sample	Males	Females	*p*
Total sample	1002	514 (51%)	488 (49%)	
Mean age (years)	10.9 ± 2.5	10.7 ± 2.5	11.0 ± 2.6	0.10
Body height (cm)	150.2 ± 16.4	150.7 ± 17.4	149.6 ± 15.3	0.31
Body mass (kg)	42.4 ± 15.8	43.2 ± 17.3	41.6 ± 14.1	0.10
BMI (kg/m^2^)	18.1 ± 3.8	18.3 ± 4.2	18.0 ± 3.5	0.19
Underweight	75 (7%)	42 (8%)	33 (7%)	0.78
Healthy	693 (69%)	330 (64%)	363 (74%)	0.12
Overweight	145 (14%)	80 (16%)	65 (13%)	0.54
Obese	84 (8%)	59 (11%)	25 (5%)	0.11
Missing	5 (2%)	3 (1%)	2 (1%)	
Residency				
Capital, county seat, or town	73%	72%	74%	0.75
Municipality, village, or farm	27%	28%	26%	0.75

**Table 2:** Participation rate of children in physical activity by gender and by weight classifications.

	Achieving MVPA > 60 min/day	Not Achieving MVPA > 60 min/day (Missing)	Club Sport	Organized Sport	Activity with Parents
Total	79%	18% (3%)	50%	41%	71%
Males	81%	16% (3%)	52%	43%	74%
Females	77%	19% (4%)	47%	39%	67%
*p*	*p* > 0.05	*p* > 0.05	*p* > 0.05	*p* > 0.05	*p* > 0.05
Underweight	80%	17%a (3%)	41%	48%	68%
Healthy	81%	16%b (3%)	53%	42%	72%
Overweight	72%	22% (6%)	42%	38%	68%
Obese	70%	29% (1%)	42%	33%c	69%
*p*	*p* > 0.05	a,b	*p* > 0.05	c	*p* > 0.05

a = significantly (*p* < 0.05) less than the obese group. b = significantly (*p* < 0.05) less than obese group. c = significantly (*p* < 0.05) less than underweight group.

**Table 3:** Odds ratios for obesity in different factors affecting physical activity in total sample and by gender.

	Odds Ratio for Obesity	CI (95%)	*p*
Achieving MVPA > 60 min/day in total sample	0.51	0.30–0.85	0.01
Males	0.44	0.24–0.83	0.01
Females	0.57	0.23–1.43	0.23
Club sport in total sample	0.64	0.41–1.02	0.06
Males	0.60	0.34–1.04	0.72
Females	0.67	0.29–1.52	0.33
Organized sport in total sample	0.67	0.42–1.09	0.10
Males	0.68	0.38–1.20	0.19
Females	0.58	0.23–1.43	0.24
Activity with parents in total sample	0.88	0.52–1.49	0.18
Males	0.74	0.39–1.39	0.35
Females	1.06	0.41–2.76	0.89
Parent achieving MVPA > 150 min/week in total sample	0.87	0.55–1.38	0.57
Males	0.89	0.51–1.56	0.69
Females	0.83	0.36–1.93	0.67
Residency (capital, county seat or town) in total sample	0.62	0.39–1.00	0.05
Males	0.50	0.29–0.89	0.01
Females	1.10	0.43–2.82	0.84

**Table 4:** Order of preferred sports and physical activities and ratios in different weight status by gender.

	**Boys**			
	**Underweight**	**Healthy**	**Overweight**	**Obese**
Club sport	Football (39%) Kayaking and Canoeing (16%) Martial arts (16%)	Football (43%) Martial arts (16%) Kayaking and Canoeing (10%)	Football (33%) Martial arts (30%) Handball (15%)	Football (33%) Martial arts (29%)
Organized sport	Football (15%) Running (15%) Swimming (10%) Martial arts (10%)	Football (30%) Swimming (11%)	Football (33%) Athletics (12%)	Football (35%) Working out in gym (20%) Table tennis (15%)
	**Girls**			
	**Underweight**	**Healthy**	**Overweight**	**Obese**
Club sport	Dancing (50%) Swimming (17%) Gymnastics (17%)	Dancing (19%) Handball (14%)	Handball (29%) Dancing (19%) Basketball (19%) Martial arts (14%)	Handball (30%) Dancing (20%)
Organized sport	Dancing (19%) Basketball (19%) Gymnastics (19%) Joga (13%)	Dancing (18%) Swimming (11%) Horse-riding (11%)	Dancing (18%)	No relevant organized activity

## Data Availability

The dataset presented in this article is not readily available because the data are part of an ongoing study.
